# Virtual Post-Intensive-Care Rehabilitation for Survivors of COVID-19: A Service Evaluation

**DOI:** 10.7759/cureus.38473

**Published:** 2023-05-03

**Authors:** Fiona Howroyd, Natacha Earle, Jonathan Weblin, David McWilliams, Jennifer Williams, Claire Storrie, Rose Brennan, Nandan Gautam, Catherine Snelson, Tonny Veenith

**Affiliations:** 1 Therapy Services, Queen Elizabeth Hospital Birmingham, Birmingham, GBR; 2 Centre for Care Excellence, University Hospitals Coventry and Warwickshire NHS Trust, Coventry, GBR; 3 Critical Care, Queen Elizabeth Hospital Birmingham, Birmingham, GBR

**Keywords:** remote, rehabilitation, follow-up, critical care, coronavirus

## Abstract

Aim: The objective of this study is to evaluate the safety, utilisation, and effectiveness of a novel, virtual rehabilitation programme for survivors of SARS‑CoV‑2 infection (COVID-19) and intensive care admission.

Methods: A service evaluation was performed. Adults admitted to a United Kingdom intensive care unit with COVID-19-induced respiratory failure and surviving hospital discharge were invited to an eight-week rehabilitation programme. The programme consisted of virtually delivered exercise classes and support groups led by critical care physiotherapists and follow-up nurses.

Results: Thirty-eight of 76 eligible patients (50%) agreed to participate, of which 28 (74%) completed the rehabilitation programme. On completion of the rehabilitation programme, there were significant improvements in exercise capacity (one-minute sit-to-stand test; 20 stands vs. 25 stands, *p *< 0.001), perceived breathlessness (Medical Research Council dyspnoea scale; 3 vs. 2 *p *< 0.001), shoulder disability (Quick Dash; 43 vs. 19 *p *= 0.001), anxiety (Hospital Anxiety Depression Scale; 4 vs. 3 *p *= 0.021), depression (Hospital Anxiety Depression Scale; 4 vs. 2.5 *p *= 0.010), and psychological distress (Intensive Care Psychological Assessment Tool; 3 vs. 2 *p *= 0.002). No adverse events or injuries were recorded during the programme.

Conclusion: It is feasible to recruit and retain survivors of COVID-19-induced respiratory failure for virtual post-intensive-care rehabilitation. It appears that the virtual rehabilitation programme is safe and improves physical and psychological morbidity.

## Introduction

Critical illness's physical and psychological sequelae are well documented and collectively known as post-intensive care syndrome (PICS) [1]. PICS is considered to affect up to 50% of intensive care unit (ICU) survivors and is recognised as a public health burden due to its lasting disability [1].

Muscle weakness and reduced exercise capacity contribute to delayed physical recovery due to an ongoing cycle of reduced physical activity and the propagation of sarcopenia and frailty [2]. Neuropsychiatric complications such as anxiety, depression, post-traumatic stress disorder, and cognitive impairment are also commonly reported [1,3]. The lasting burden of PICS leads to an impairment in quality of life, exacerbation of long-term conditions, and increased healthcare utilisation for patients and their relatives [1-3].

Acute respiratory failure following a SARS‑CoV‑2 infection (COVID-19) has resulted in a sudden surge in ICU admissions worldwide [4]. The physical and psychocognitive consequences specifically associated with COVID-19 have been recognised, but not yet fully understood in relation to PICS [5-6]. Patients with severe COVID-19 are known to require extended periods of mechanical ventilation, deep sedation, neuromuscular blocking agents, and prolonged intensive care admissions, all of which are common risk factors for the development of PICS [7]. Other risk factors associated with PICS include comorbidities such as diabetes, hypertension, and chronic lung disease, a similar profile to that seen in severe cases of COVID-19 [7].

Rehabilitation has been recognised as an essential component in the recovery from PICS and COVID-19 [8-10]. However, many rehabilitation and outpatient services have faced significant disruption during the pandemic [10-11]. Urgent work must ensure appropriate rehabilitation services are delivered, and novel ways of safe working should be established and protected [10-11].

The post-intensive-care follow-up service at our institution formerly consisted of a weekly rehabilitation class and support group led by ICU physiotherapists and ICU follow-up nurses in the hospital's outpatient department. Due to the demands of the pandemic, the follow-up service was suspended to enable the repurposing of staff and resources for frontline emergency services. Although some outpatient services have since reopened, national laws regarding social distancing and infection-control procedures have restricted face-to-face group activities.

To meet the needs of a rapidly increasing post-intensive-care population amidst the unprecedented circumstances of a pandemic, services were required to adapt and work virtually [10-11]. The concept of telerehabilitation has been shown to be possible for COVID-19 patients, but not specifically for COVID-19 survivors of critical care admission [12-13]. In July 2020, the critical care follow-up team re-established and developed an innovative, virtual post-intensive care rehabilitation programme.

This service evaluation aimed to assess the safety, utilisation (recruitment, retention, and adherence), and potential impact on the recovery from physical and neuropsychiatric sequelae of an innovative virtual post-intensive-care rehabilitation programme for survivors of COVID-19.

This article was previously presented as a meeting abstract at the 2021 European Society of Intensive Care Medicine (ESICM) Lives Conference on October 4, 2021, and the 2021 Association of Chartered Physiotherapists in Respiratory Care (ACPRC) ACPRC Virtual Conference on April 23, 2021 [14-15].

## Materials and methods

Design

This was a single-centre service evaluation and has therefore been reported in accordance with Revised Standards for Quality Improvement Reporting Excellence (SQUIRE) guidance [16].

Ethical considerations

This single-centre (University Hospitals Birmingham NHS Foundation Trust (UHB)) service evaluation used anonymised linked databases (Clinical Audit and Registration Management System [CARMS] Identification Number: CARMS-17954) with a waiver of ethical approval according to the national guidance [17]. We used routinely collected data, conducted within the clinical audit framework; patients were not contacted outside their routine clinical care.

Setting

A major tertiary hospital in Birmingham, United Kingdom (The Queen Elizabeth Hospital Birmingham (QEHB)), with one of the largest co-located ICUs in Europe. Between March 2020 and April 2020, the ICU at QEHB admitted 177 patients with COVID-19, of whom 110 survived ICU discharge [7].

Sample

Consecutive adult patients (≥18 years of age) who were admitted to the ICU with a confirmed diagnosis of COVID-19 between March 2020 and April 2020 and survived to hospital discharge were identified by a senior ICU physiotherapist. Patients were excluded if they had unstable medical conditions contraindicating their safety to exercise, a severe neurological injury impeding their ability to cooperate with rehabilitation, or an inability to provide informed consent for the programme as required by our hospital.

After hospital discharge, patients were invited to participate via telephone by a senior ICU physiotherapist. If patients declined to participate, the primary reason was recorded. Baseline data, including demographics, ventilation days, and length of stay, were obtained from patient notes and electronic health databases.

Intervention

Patients were invited to participate in an eight-week post-intensive-care rehabilitation programme. The programme consisted of a once-weekly exercise class followed by a support group, completed virtually via video call using Microsoft Teams. Patients were able to complete the programme in their own homes using either a smartphone, tablet device, laptop, or computer, with a group of up to six patients on each call. Verbal and written patient information was provided, including the risks and benefits [18].

Exercise Component

A structured, interval exercise approach was adopted, consisting of a warm-up, 20 minutes of circuit-based exercises, and a cool-down, delivered by the class physiotherapist [19]. The programme was designed so patients could complete the exercises from a chair and therefore remain visible on the video screen. Ten exercises were completed in each circuit, with one minute for each exercise completed twice. A combination of upper- and lower-limb exercises aiming to improve strength and cardiovascular fitness were included [19]. Patients were advised of four different intensity levels for each exercise (Appendix 1, Table 3) [18]. Patients were advised to titrate exercise intensity based on their perceived breathlessness, aiming for a score of 3-4 on the modified Borg breathlessness scale (Appendix 2, Table 4) [18-20]. An online patient information booklet was provided to each participant, including written instructions and photographs of each exercise, in addition to the Borg breathlessness scale [18].

Support Group Component

Following the exercise component, patients were invited to participate in a support group. The structure of these sessions is based on recommendations from the patient support charity 'ICU Steps' [21]. In line with these recommendations, the groups were supported by a senior nurse with experience in ICU follow-up and a senior critical care physiotherapist. The combination of a nurse and physiotherapist with experience in managing patients during and following critical illness ensures appropriate expertise in both physical and neuropsychiatric recovery. The role of the healthcare professionals was to support new participants and facilitate discussion. Patients were given the opportunity to share their experiences with their ICU recovery. Educational topics such as delirium, sleep, pacing advice, and expectations of recovery were discussed.

Primary outcome measures

The primary outcomes assessed the utilisation of the virtual rehabilitation programme; including recruitment, retention, and adherence rates. Recruitment was defined as the percentage of eligible patients who attended. Retention was defined as the number of patients who completed rehabilitation. Adherence was defined as completing a minimum of 75% of the available virtual rehabilitation sessions. All adverse events were recorded.

Secondary outcome measures

Secondary outcomes assessed the impact of the virtual rehabilitation programme on patient clinical outcomes (Appendix 3, Table 5). Outcomes were assessed by the class physiotherapist during an individual video-call consultation before commencing the programme and within one week of its completion. Physical outcomes included the one-minute sit-to-stand test (1MSTS), the Medical Research Council breathlessness scale (MRC), and the Quick Dash Upper Limb questionnaire (QD). Non-physical outcomes included the Hospital Anxiety and Depression Score (HADS), the Intensive Care Psychological Assessment Tool (IPAT), and the perceived health rating of the Euro-Qol 5 dimension quality of life questionnaire (EQ5D).

Statistical analysis

Data for all outcome measures were assessed using IBM SPSS 26 software (IBM Corp., Armonk, NY). The characteristics of the patient cohorts who attended and did not attend were summarised, with continuous variables reported as means ± standard deviations (SDs) where normally distributed and medians and interquartile ranges (IQRs) reported otherwise. Comparisons between patients who attended and did not attend were then performed using Mann-Whitney U tests for ordinal or continuous variables and chi-squared tests for nominal variables. Data for all outcomes post-intervention were assessed for normality using the Sharipo-Wilk test, and non-parametric tests were used as appropriate. Continuous variables were reported as medians and IQRs, and the Wilcoxon matched-pairs signed rank test was used to assess for statistical significance, with p<0.05 deemed to be indicative of statistical significance throughout.

## Results

**Figure 1:**
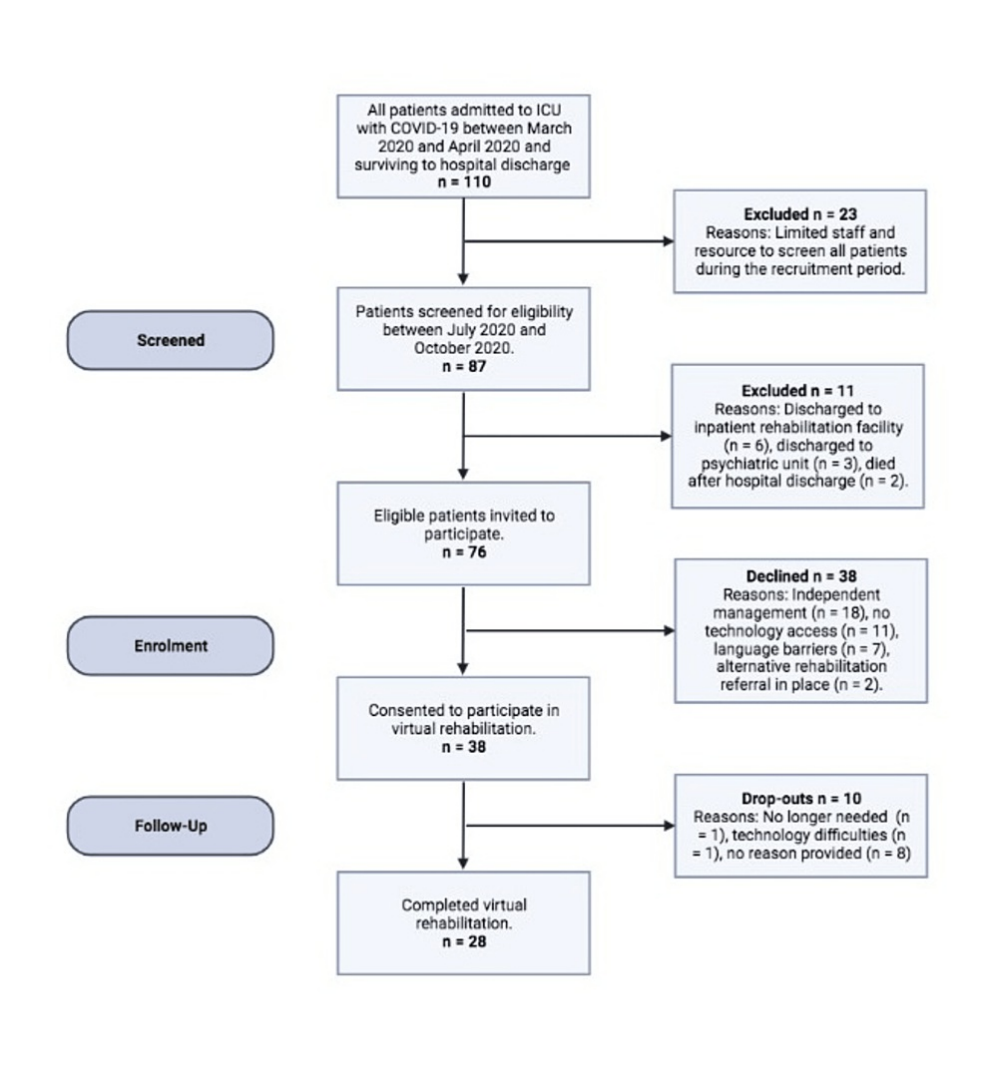
Diagram to demonstrate recruitment and retention to virtual rehabilitation. ICU: intensive care unit; COVID-19: coronavirus disease 2019 infection.

Eighty-seven patients out of 110 patients (79%) who were admitted to the ICU at QEHB with COVID-19 between March and April 2020 and survived hospital discharge were screened for their eligibility for virtual post-intensive-care rehabilitation during a three-month period (Figure 1). Seventy-six of those screened (87%) were eligible for rehabilitation, with 38 patients consenting to the programme (50%). The reasons for declining rehabilitation (n=38) were independent self-management (47.4%), lack of technology access (the patient did not have access to a smartphone, tablet device, laptop, or computer) (28.9%), language barrier (18.4%), and other rehabilitation programmes in place (5.3%). Of the 38 who agreed to participate, 28 completed the programme (74%) (Figure 1). Of those that completed, 82% adhered (n = 23); quantified as attending 75% of planned rehabilitation sessions. Patients attended rehabilitation for a mean of 79 days following discharge from the hospital. No serious adverse events or injuries were recorded during the programme.

The baseline characteristics of patients invited to the post-intensive-care rehabilitation programme can be found in Table 1. Patients who attended virtual rehabilitation were predominantly male (79%) with a mean age of 54 years (standard deviation [SD] 11). There were no statistical differences in demographics between those who completed the virtual rehabilitation programme and those who declined (Table 1).

**Table 1:** Demographic and clinical characteristics of patients who completed virtual rehabilitation and those who declined (n = 66). SD: standard deviation; CFS: clinical frailty scale; BMI: body mass index; ICU: intensive care unit.

Demographics	Completed (n = 28)	Declined (n = 38)	p-value
Mean age (SD)	54 (11)	54 (12)	0.816
Gender; male/female	22 (79%)/6 (21%)	28 (74%)/10 (26%)	0.647
Median CFS on admission	2 (2–2.3)	2 (1–2)	0.213
Mean BMI (SD)	32 (6)	31 (6.3)	0.793
Median ICU length of stay	21 days (18.75–27.75)	21.5 days (13.5–28)	0.421
Median hospital length of stay	33 days (30–46)	38 days (19–38)	0.765
Median ventilator days	20 days (16–24)	17.5 days (10–26.5)	0.381

Following completion of the virtual rehabilitation programme, there was a statistically significant improvement in all physical outcomes (Table 2). After rehabilitation, there was an observed increase in exercise capacity (1 MSTS 20 stands vs. 25 stands, p<0.001) and a reduction in perceived breathlessness (MRC 3 vs. 2, p<0.001). Shoulder disability scores were also reduced after participation in rehabilitation (QD 43.18 vs. 19.31, p=0.001).

**Table 2:** Results of physical and non-physical outcomes pre and post rehabilitation (n = 28). 1MSTS: one-minute-sit-to-stand test; QD: Quick Dash Shoulder Disability questionnaire; MRC: Medical Research Council dyspnoea scale; HADS-A: Hospital Anxiety and Depression Score for Anxiety; HADS-D: Hospital Anxiety and Depression Score for Depression; IPAT: Intensive Care Psychological Assessment Tool; the perceived health rating of the Euro-Qol 5 dimension quality of life questionnaire (EQ5D); IQR: inter-quartile range.

Outcomes	Pre-rehabilitation (median)	Post-rehabilitation (median)	p-value
1 MSTS	20 stands (IQR 15–27)	25 stands (IQR 19–31)	<0.001
QD	43.18 (IQR 7.86–53.95)	19.31 (IQR 6.80–37.49)	0.001
MRC	3 (2–3)	2 (1–2)	<0.001
HADS-A	4 (IQR 2–8)	3 (IQR 1–6.75)	0.021
HADS-D	4 (IQR 2–7)	2.5 (IQR 1–5.5)	0.010
IPAT	3 (IQR 1.25–6.75)	2 (IQR 1–4)	0.002
EQ5D	60 (IQR 50–80)	76 (IQR 65–90)	0.001

There was also a statistically significant improvement in all non-physical outcomes (Table 2) (HADS anxiety: 4 vs. 3 p=0.021; HADS depression: 4 vs. 2.5 p=0.010; IPAT: 3 vs. 2 p=0.002). However, median scores for anxiety (HADS anxiety), depression (HADS depression), and psychological distress (IPAT) were suggestive of no clinical psychological symptoms prior to commencing rehabilitation. Further analysis of the non-physical data showed nine of the 28 (32%) participants had clinical symptoms of anxiety (HADS anxiety score ≥ 8) prior to rehabilitation, reducing to six participants after rehabilitation. Six (21%) participants had clinical symptoms of depression prior to rehabilitation (HADS depression score ≥ 8), reducing to three post-rehabilitation. Six (21%) participants had clinical symptoms of psychological distress prior to rehabilitation (IPAT score > 7), reducing to one post-rehabilitation. Perceived health ratings also showed significant improvement following rehabilitation (EQ5D 60 vs. 76, p=0.001).

## Discussion

We assessed the safety, utilisation (recruitment, retention, and adherence), and impact on the recovery from physical and neuropsychiatric sequelae with an innovative virtual post-intensive-care rehabilitation programme in survivors of COVID-19. To the best of our knowledge, this is the first known report of innovative, virtually delivered post-intensive-care follow-up specifically for survivors of severe COVID-19 requiring ICU admission.

This service evaluation has demonstrated that it is feasible to recruit survivors of an ICU admission with severe COVID-19 to a virtual, post-intensive-care rehabilitation programme. During the three-month recruitment period, 79% of patients were approached and offered rehabilitation. Patients were approached an average of 79 days post-hospital discharge, in accordance with national guidelines, which advise follow-up at two to three months [8]. Twenty-three patients were not offered rehabilitation due to staffing constraints. Patients found the experience and understanding of ICU clinicians valuable; however, it is recognised that the service requires protected resources and funding to meet the demands of the COVID-19 pandemic. A previous survey identified that intensive-care follow-up services are notoriously underfunded and only provided by 27% of UK intensive care units, with less than 10% offering follow-up in the form of structured exercise [22]. Further work is required to ensure follow-up services are equitable and accessible to all ICU survivors.

Of those approached during the period of the service evaluation, 50% participated in the virtual post-intensive-care rehabilitation programme. The primary reason for declining was that rehabilitation was not required due to independent self-management. This may be reflective of a working-age cohort (mean age 54 years) and pre-admission Clinical Frailty Score (median 2 = well); however, in our study, the demographics of those who attended and those who declined showed no significant differences. The physical and non-physical outcomes of those who declined rehabilitation were not investigated in this study, yet previous literature suggests that PICS affects 50% of critical care survivors [1,8]. Predictors of long-term morbidity in survivors of COVID-19 are currently unknown.

Of those who attended, we found 73.6% retention (defined as completion of the programme) and 82% adherence (defined as attending a minimum of 75% of rehabilitation sessions) to an eight-week programme; demonstrating patient acceptability of the programme. The virtual nature of the programme has enabled accessibility to rehabilitation and the ability to overcome the challenges of social restrictions and infection control procedures during the COVID-19 pandemic. However, it is important that rehabilitation services do not discriminate against those who do not have access to technology. Almost one-third of patients declined participation due to a lack of technology access, and 18.2% due to language barriers. Further work is required to ensure rehabilitation and post-intensive care follow-up are accessible to all patients, expanding to all socioeconomic and ethnic regions.

The programme was found to be safe, with no adverse or safety events recorded. The participants who completed the rehabilitation programme demonstrated improvements in all physical and non-physical outcomes. Despite being delivered virtually, with patients self-directing their exercise intensity based on perceived breathlessness, the rehabilitation programme remained effective. This supports existing work that virtual rehabilitation is feasible and effective, yet this is the first known report of virtual rehabilitation specifically for survivors of COVID-19 following critical care admission [12-13,23-24].

The rehabilitation programme aimed to improve physical fitness through an interval training approach. Baseline data identified poor levels of exercise capacity prior to rehabilitation, with the mean 1MSTS score for the group equivalent to the 2.5th percentile for healthy 65- to 69-year-olds, despite a mean age of 54 years [25]. Following completion of the programme, patients demonstrated a significant improvement in exercise capacity (1 MSTS 20 vs. 25, p<0.001). However, it is important to note that although the 1MSTS results improved, the scores remained below the 2.5th percentile for the 50-54 age groups [25]. The 1MSTS is a pragmatic measure of exercise capacity during a virtual assessment [26]. In chronic obstructive pulmonary disease cohorts, the 1MSTS has been found to be a reliable and valid measure of exercise capacity, comparable with the six-minute walk test; a measure that has been utilised in previous ICU research [27].

In addition to physical fitness, the programme also aimed to improve upper-limb strength. Shoulder impairment and its associated functional disability are common in survivors of critical illness [28]. Pronounced upper limb deficits have been found in COVID-19 survivors due to injuries associated with prone positioning [29]. Our cohort demonstrated severe upper limb dysfunction at baseline. On completion of the programme, patients showed a significant reduction in shoulder dysfunction (QD 43.18 vs. 19.31, p=0.001).

In contrast to previous ICU literature, overall, our cohort demonstrated normal scores for anxiety, depression, and psychological distress [1,3]. Despite this, psychological symptoms continued to show significant improvement following the completion of the programme. Psychological morbidity has been known to influence adherence to medical treatment in other populations; therefore, results may only be reflective of those who participated [30]. At the point of contact, many patients reported gratitude for their survival and returned home to their relatives after a period of separation during their hospital admission. This may explain the low levels of psychological symptoms in our cohort. A longitudinal, qualitative exploration of the psychological recovery of COVID-19 survivors should be considered, exploring the unique experiences of COVID-19 patient groups and potential long-term impacts.

Limitations

There are several limitations to this service evaluation. First, this is a non-randomised trial that does not have a control group for comparison. We recognise that this reduces the validity of the study findings as the results may be subject to temporal changes, acknowledging that activity levels between hospital discharge and follow-up were not monitored and that the advice given to patients by ward physiotherapists was not standardised. The results are also open to bias as they only reflect those who consented to participate and are therefore representative of a cohort that is motivated and engaged with rehabilitation. As this service evaluation did not collate qualitative data, the results do not account for the experiences, needs, or preferences of patients. Participant numbers were small, and the service evaluation was performed at a single centre; therefore, results may not be representative of the wider population.

## Conclusions

The COVID-19 pandemic has placed overwhelming demand on healthcare services and has subsequently led to the repurposing of resources and staff to bolster frontline emergency departments. As we face the secondary impacts of the pandemic, the optimisation of recovery as a therapeutic objective should be prioritised over patient survival alone. For many survivors of critical illness, their discharge from the ICU is the start of an uncertain journey, facing numerous physical and non-physical limitations. It is essential that critical care rehabilitation and follow-up services are protected in order to optimise patient recovery and quality of life after critical illness.

This service evaluation demonstrates that with protection and prioritisation of rehabilitation, post-intensive-care follow-up services can be adapted using innovative and virtual methods. This is the first known report of virtual post-intensive-care rehabilitation for survivors of COVID-19, providing accessible critical care follow-up despite the restrictions faced by healthcare services during the pandemic. This service evaluation has demonstrated that it is feasible to recruit and retain intensive care survivors for virtual rehabilitation and that the programme is safe and effective. For those who attended the virtual post-intensive-care rehabilitation programme, there was a significant improvement in all physical and non-physical outcomes. Further research, including an appropriately powered multi-centre randomised clinical trial with longitudinal follow-up, is required to further validate these findings.

## Appendices

Appendix 1

**Table 3:** Exercise component of the rehabilitation class. Lv1: level 1 exercise; Lv2: level 2 exercises; Lv3: level 3 exercises; Lv4: level 4 exercises [18].

Exercise	Exercise Levels
1. Sit to Stand. Start by sitting on your chair. Stand up and then sit back down. Repeat for one minute.	Lv1: x10 stands, using arms to support; Lv2: x20 stands, light use of arms to support; Lv3: x20 stands, without use of arms; Lv4: x20 stands, holding a weight.
2. Arm Raises. Start with your hands by your side. Lift your arms out to the side, and towards the ceiling as far as you can. Return your arms to your side. Continue for one minute.	Lv1: Arm raises whilst seated; Lv2: Arm raises in standing - hold onto a chair if required for balance, alternating each arm; Lv3: Arm raises in standing - holding a weight; Lv4: Arm raises in standing - holding a weight and marching on the spot.
3. Side Steps. In standing, step out sideways and then back in with your right leg. Repeat with your left leg. Continue for one minute.	Lv1: Side step alternate legs in sitting; Lv2: Side steps in standing holding onto a chair; Lv3: Side step, bringing both arms to shoulder height; Lv4: Side step bringing both arms to shoulder height, holding weights.
4. Marching. March on the spot for one minute.	Lv1: Seated marching; Lv2: Standing marching whilst holding onto chair; Lv3: Standing marching with no arm support; Lv4: Standing marching holding weights.
5. Ball around the body. Pass a ball around your body clockwise for 30 seconds, then anticlockwise for 30 seconds. If you do not have a ball, you could use a cushion.	Lv1: Pass a small ball/weight whilst seated; Lv2: Pass a small ball in standing; Lv3: Pass a weighted ball/weight in standing; Lv4: Pass a weighted ball/weight in standing whilst marching on the spot.
6. Squats. Hold onto a support/chair as required. Keep your back straight, bend your knees into a squat position. Straighten your knees and repeat for one minute.	Lv1: Squat to 45° slowly, holding onto a chair; Lv2: Squat to 70° slowly, holding onto a chair; Lv3: Squat to 70° without holding onto support; Lv4: Squat to 70° holding a weight.
7. Ball Lift. Hold a ball in both hands. Lift the ball towards the ceiling as far as you can. Bring the ball back down to your chest. Repeat for one minute.	Lv1: Lift small ball in sitting; Lv2: Lift small ball in standing; Lv3: Lift weighted ball in standing; Lv4: Lift weighted ball whilst spot marching.
8. High Knee Marching. Hold onto a support/chair if required. March knees up and down, lifting knees up to 90°. Repeat for one minute.	Lv1: In sitting, march knees up and down; Lv2: Hold onto chair in standing, march on the spot lifting knees; Lv3: March on the spot with high knees, without support/holding onto chair; Lv4: March on the spot with high knees, holding weights. Increase your speed as you feel able.
9. Bicep Curls. Hold onto hand weights and slowly bend and straighten your right elbow. Repeat with your left side. Continue for one minute.	Lv1: Complete in sitting with small weight; Lv2: Complete in standing with small weight, holding onto chair as required; Lv3: Complete in standing, holding medium weight, while marching on the spot; Lv4: Complete in standing, holding heavy weight while marching on the spot.
10. Heel raises. Stand holding onto a support/chair if required, lift heels off the ground, bringing yourself onto tip- toes. Slowly lower back down. Repeat for one minute.	Lv1: Heel raises in sitting; Lv2: Heel raises in standing holding onto a chair; Lv3: Heel raises in standing without support; Lv4: Heel raises in standing holding a weight.

Appendix 2

**Table 4:** Modified Borg breathlessness scale. [20].

Modified Borg scale	
The scale below helps you record how breathless you feel. It is important that you are aware of how to record your breathlessness as this will help to show how you are progressing. It is normal to feel more breathless when exercising, reaching a level of 3 or 4 on the scale below.	
Scale	Severity of breathlessness
0	Nothing at all
0.5	Very, very slight
1	Very slight
2	Slight
3	Moderate
4	Somewhat severe
5	Severe
6	
7	Very severe
8	
9	Very, very severe
10	Maximal

Appendix 3

**Table 5:** Secondary outcome measures. 1MSTS: one-minute-sit-to-stand test; QD: Quick Dash Shoulder Disability questionnaire; MRC: Medical Research Council dyspnoea scale; HADS: Hospital Anxiety and Depression Score; IPAT: Intensive Care Psychological Assessment Tool; EQ5D: the perceived health rating of the Euro-Qol 5 dimension quality of life questionnaire.

Outcome measure	Brief overview
1MSTS	A measure of exercise capacity. The patient is asked to stand up and sit down as many times as they can in one minute whilst being observed during a video consultation. The number of stands completed is recorded.
MRC	A self-reported rating scale of breathlessness to measure perceived respiratory disability. The patient is asked to score themselves on a scale of 0 to 5 to grade the extent to which breathlessness affects their mobility.
QD	A self-reported questionnaire of musculoskeletal disorders of the upper limb. The QD uses 11 items to score physical function and symptoms (including pain) in order to indicate disability and severity.
HADS	A self-reported questionnaire to assess psychological distress by measuring symptoms of anxiety (HADS Anxiety) and depression (HADS Depression). The questionnaire uses fourteen self-rating scales, with seven items for anxiety and seven for depression. Anxiety and depression are score separately. A score of 0-7 is considered normal, 8-10 borderline abnormal and 11-21 abnormal.
IPAT	A self-reported questionnaire to assess psychological distress and the risk of future psychiatric morbidity in critical care patients. The questionnaire includes 10 items, including symptoms of impaired sleep and hallucinations. The sum of scores for each item provides a total IPAT score out of 20. A total score of 7 or more indicates a patient who is at risk of psychological distress.
EQ5D	The EQ5D is a measure of health-related quality of life. One component of this was utilised; the patient is asked to rate their perceived health on the day of the assessment on a scale of 0 to 100.
